# Molecular Mechanism of Fucoidan Nanoparticles as Protector on Endothelial Cell Dysfunction in Diabetic Rats’ Aortas

**DOI:** 10.3390/nu15030568

**Published:** 2023-01-21

**Authors:** Giftania Wardani, Jusak Nugraha, Rochmah Kurnijasanti, Mohammad Rais Mustafa, Sri Agus Sudjarwo

**Affiliations:** 1 Doctoral Program of Medical Science, Faculty of Medicine, Universitas Airlangga, Surabaya 60115, Indonesia; 2 Program Study of Pharmacy, Faculty of Medicine, Hang Tuah University, Surabaya 28125, Indonesia; 3 Department of Clinical Pathology, Dr. Soetomo Hospital, Universitas Airlangga, Surabaya 60115, Indonesia; 4 Department of Pharmacology, Faculty of Veterinary Medicine, Universitas Airlangga, Surabaya 60115, Indonesia; 5 Department of Pharmacology, Faculty of Medicine, University of Malaya, Kuala Lumpur 50603, Malaysia

**Keywords:** fucoidan nanoparticle, antioxidant, endothelial cell, diabetes

## Abstract

Antioxidants have an important role in protecting against diabetes complications such as vascular endothelial cell damage. Fucoidan has strong antioxidant properties, therefore the aim of this study was to investigate the protective mechanism of fucoidan nanoparticles through the pathway of antioxidant activity against streptozotocin-induced diabetic aortic endothelial cell dysfunction in rats. Fucoidan nanoparticles are made utilizing high-energy ball milling. This research consists of five groups, namely: control rats, rats were administered aquadest; diabetic rats, rats were administered streptozotocin (STZ); fucoidan nanoparticle rats, rats were administered STZ and fucoidan nanoparticles. Aortic tissue was collected for the evaluation of ROS (reactive oxygen species), Malondialdehyde (MDA), superoxide Dismutase (SOD), Glutathione Peroxidase (GPx), Nuclear factor erythroid-2-related factor 2 (Nrf2), Nitric Oxide (NO), cyclic Guanosine Monophosphate (cGMP), relaxation response of acetylcholine (Ach), and the diameter of the aorta. The size distribution of the fucoidan nanoparticles was 267.2 ± 42.8 nm. Administration of fucoidan nanoparticles decreased the levels of ROS and MDA, and increased the levels of SOD, levels of GPx, Nrf2 expression, NO levels, cGMP expression, the relaxation response of Ach, and lumen diameter of the aorta, which are significantly different when compared with diabetic rats, *p* < 0.05. In this study, we concluded that the mechanism pathway of fucoidan nanoparticles prevents aortic endothelial cell dysfunction in diabetic rats through antioxidant activity by reducing ROS and MDA and incrementing SOD levels, GPx levels, and Nrf2 expression. All of these can lead to an elevated relaxation response effect of Ach and an increase in the lumen diameter of the aorta, which indicates a protective effect of fucoidan nanoparticles on aortic endothelial cells.

## 1. Introduction

One of the markers of diabetes mellitus is hyperglycemia, which can cause complications in various organs such as the liver, kidneys, nerves, eyes, and heart, as well as endothelial dysfunction of blood vessels. This is due to an impaired pancreas to secrete insulin or because of insulin resistance [1,2]. Diabetes mellitus is one of the major risk factors for cardiovascular disease, which is the most common cause of death in diabetic complications. In addition to the well-recognized microvascular diabetic complications, such as nephropathy, retinopathy, and neuropathy, there is a growing epidemic of macrovascular diabetic complications, including coronary artery disease, peripheral artery disease, and vascular endothelial cell dysfunction. Endothelial cell dysfunction can be due to glycometabolic disorder, genetics, oxidative stress, inflammatory cytokines, and apoptosis, which are the main causes of cardiovascular diseases such as atherosclerosis. Oxidative stress plays a crucial role in causing endothelial cell dysfunction in diabetic complications [3,4,5]. Oxidative stress can be caused by the overproduction of reactive oxygen species such as hydroxyl radicals (OH-), superoxide anions (O_2_^−^), and hydrogen peroxide (H_2_O_2_), and a lower antioxidant defense system such as SOD, GPx, and catalase. Excessive ROS formation occurs through the polyol pathway, increased production of advanced glycation end-products (AGEs), the hexosamine pathway, and protein kinase C activation, which can lead to diabetic complications [6,7,8]. Increased ROS levels in diabetes can lead to decreased production of SOD, GPx, and catalase. The ROS will oxidize polyunsaturated fatty acids (PUFA) that are abundant in cell membranes to form MDA. This MDA can be used as an indicator of the cell damage caused by free radicals under conditions of oxidative stress [9,10].

Increased ROS and decreased antioxidants can also occur in diabetic rat models injected with intraperitoneal streptozotocin. Previous studies revealed that administration of streptozotocin can reduce the expression of Nrf2, which is the main regulator for the formation of antioxidants; so lower SOD, GPx, catalase, and higher MDA in the aortic tissue of diabetic rats [11,12]. It has been reported that increasing ROS can inactivate Nrf2, resulting in a decrease in Nrf2 expression. This increase in ROS will cause cysteine damage in Keap1, disrupt the Keap1-Cul3 ubiquitination mechanism, and mediate Nrf2 degradation by the 26S proteasome so that Nrf2 has a short half-life, which can result in decreased expression of Nrf2 [13,14]. Animal experiments have shown that the Nrf2/Keap1 system is an important defense pathway for protecting against cell damage under conditions of oxidative stress. Decreased expression of Nrf2 can reduce the expression of cytoprotective antioxidants (SOD, GPx, and Cat) in transgenic mouse cells and exacerbate oxidative cell damage [15,16].

In a diabetic rat model, injection of STZ increased ROS and caused endothelial cell dysfunction, which resulted in decreased formation of NO and cGMP in aortic tissue. Therefore, natural products that have antioxidant effects can be used to prevent endothelial cell damage in diabetes [17,18,19]. The antioxidants from natural products such as Cinnamomum cassia and Heterotrigona itama could be used as protection against endothelial cell dysfunction in diabetic complications, as they are shown to decrease ROS and increase the NOS, NO, and cGMP in rat aortic tissue that was given streptozotocin intraperitoneally [20,21]. Fucoidan derived from seaweed is reported to have strong antioxidant activity. Recently, many researchers have conducted studies on the potential and pharmacological activities of fucoidan, such as anti-inflammatory, antibacterial, anti-apoptotic, immunostimulant, anti-atherosclerotic, anti-diabetic, and antioxidant [22,23,24,25].

In recent times, there have been many studies using nanotechnology to make nanoparticles of natural materials. Natural-product nanoparticles are considered to have an important role in treating and preventing diseases, both in humans and animals. Natural-product nanoparticles based on the nanoparticle size scale are more beneficial for increasing solubility, absorption, bioavailability, stability, distribution, and effectiveness in prevention and treatment [26,27].

Fucoidan has anti-diabetic and antioxidant effects, therefore, this study aims to make preparations of fucoidan nanoparticles that are used to prove the molecular mechanisms of fucoidan nanoparticles, to protect against endothelial cell dysfunction in rats induced by streptozotocin.

## 2. Materials and Methods

### 2.1. Fucoidan Nanoparticles Preparation

Fucoidan nanoparticles are made utilizing the high-energy ball milling method, which was carried out according to the instructions of the nanomachine manufacturer [28]. Furthermore, size of fucoidan nanoparticles was characterized by dynamic light scattering (Horiba LA 900, Kyoto, Japan).

### 2.2. Experimental Methods of Animals

Body weight of Wistar rats was 280–300 g; rats were obtained from LPPT, Universitas Gajah Mada, Indonesia. Prior to the experiment, rats were acclimatized for one week in plastic cages with a 12 h light/dark cycle at 26 ± 2 °C. All rats were given commercial pellets and drinking water ad libitum. All procedural experiments were approved by the Medical Ethics for Research in Preclinical trials of Hang Tuah University of Medical Sciences (approval number: No. 283/FK. UHT/II/2022).

### 2.3. Model of Diabetic Rat

After the rats fasted for 12 h, then the rats were given intraperitoneally a single dose of streptozotocin of 55 mg/kg BW. Streptozotocin was dissolved in citrate buffer 0.1 M (pH 4.5). The rat blood was checked for blood sugar levels with Accu-check glucometer (Roche Diagnostic) 3 days after the injection of streptozotocin. Rats with blood sugar levels > 200 mg/dL were used in the experiment.

### 2.4. Experimental Design

The rats were randomly divided into five groups (*n* = 10 in each group): control rats, rats were administered aquadest; diabetic rats, rats were injected intraperitoneally with a single dose of streptozotocin of 55 mg/kg BW; fucoidan nanoparticles rats, rats were injected with streptozotocin 55 mg/kg BW intraperitoneally and then after 3 days rats were administered fucoidan nanoparticles at doses of 75, 150, and 300 mg/kg BW, orally, once a day, for 75 days. At the end of the research, all rat aortas from various treatments were collected to evaluate MDA, SOD, GPx, Nrf2, NO, cGMP, relaxation response of Ach, and diameter of the aortic lumen.

### 2.5. Measurement of ROS in Aortic Endothelial Cells

Rats is anesthetized with ketamine intraperitoneally and the chest was opened to remove the aorta with dissection scissors. Then, the aorta was inserted into cold (sterile) PBS, and the container was moved to laminar airflow. A 1 mL syringe, equipped with a 25 G needle, was inserted into one end of the aorta, and the aorta was gently flushed with cold PBS to remove blood. The aorta was transferred to 20% fetal bovine serum (FBS)-DMEM medium. The aorta was cut into 1 mm rings and opened using sterile micro-dissection scissors to harvest endothelial cells of approximately 8–10 rings per aorta.

The using fluorescent probe, DCFH-DA was used to examine the levels of ROS in aortic endothelial cells (Sigma Aldrich; Merck, Germany). Endothelial cells were rinsed with PBS and added to 10 µM DCHF-DA for 30 min at 37 °C in darkness. The fluorescent signal of DCHF-DA was analyzed by flow cytometry (BD Vioscience, Franklin Lakes, NJ, USA) with Cell Quest Software version 5.1.

### 2.6. Measurement of SOD and GPx Levels of Aortic Tissue

Fifty milligrams of aortic tissue was washed five times with phosphate buffer saline (PBS) until clean. Then, sample was added to 0.5 mL of PBS and crushed with a mortar. Next, it was centrifuged at 10,000 rpm for 10 min. SOD levels in the supernatant were measured with a rat SOD ELISA kit (Bio-Vision K335–100, Milpitas, CA, USA); GPx was measured with a rat GPx ELISA kit (Bio-Vision K 762–100, Milpitas, CA, USA), according to the manufacturer’s protocol. Sample was put into a microplate and incubated for 90 min at 37 °C. Afterward, it was incubated with the biotinylated antibody at 37 °C for 60 min. Then, rinsed with PBS three times and incubated at 37 °C for 30 min with working solution of avidin–biotin complex with PBS. Next, the sample was incubated with TMB color development at 37 °C for 30 min. Finally, the TMB stop solution was added and the OD value was read by microplate reader at 450 nm. SOD and GPx levels were expressed in U/mg protein.

### 2.7. Extraction of RNA and RT-PCR Analysis for Nrf2 Detection

Trizol solution was used in isolating total RNA from rat kidneys, in addition, it was re-suspended in 50 L of water with pyrocarbonate and at 80 °C it was stored. Furthermore, Promega reverse transcription was used to synthesize cDNA for Nrf2 detection (5); 5- TTGTAGATGACCATGAGTCGC-3 for forward and 5- TGTCCTGCTGTATGCTGCTT-3 for reverse are the specific primers used in this case. This is because mouse-actin mRNA amplification controls in each sample use a primer, the primer used for forward is 5-GAGGCTCAGAGCAAGAGAGG -3, while for retreat they use 5 TGACATCTCGCACAATCTCC-3. All polymerase chain reactions were brought out by tagging DNA polymerase (Life Technology Inc., Monza, Italy) with 200 ng cDNA, which were for 30 amplification cycles each; consisting of one minute for the denatured phase at 94 °C, 30 °C; seconds for phase annealing at 65 °C; and the elongation in 1 min at 72 °C. Furthermore, on the 2% agarose gel, the polymerase chain reaction product was amplified and then separated and the band was visualized using ethidium bromide. Scion Image software was used with densitometry to measure the density of the Nrf2 expression band (Scion Corporation, Frederick, MD, USA).

### 2.8. Measurement of NO Levels in Aortic Tissue

Griess reagent was used to evaluate NO levels in aortic tissue. For this, 100 µL homogenate of aortic tissue was mixed with 100 µL Griess reagent (1% Sulfanilamide and 0.1% Naphthylethylenediamine Dihydrochloride in 5% phosphoric acid) and incubated at room temperature for 10 min. The absorbance was measured by microplate reader at 540 nm. NO levels were expressed in terms of µg equivalence of NANO_2_/mg protein.

### 2.9. Immunohistochemical Staining of MDA and cGMP in Aortic Tissues

The cGMP was determined by immunohistochemistry. To suppress endogenous peroxide, 4 µm aorta tissue slices were deparaffinized and then exposed to hydrogen peroxide at 37 °C for 10 min. Furthermore, a tris-buffered salt solution was added, and temperature was maintained at 37 °C for 30 min. They are then washed in 3× PBS and incubated with a secondary antibody from Thermofisher Scientific for 30 min at room temperture. The immunostaining was visualised using 3,3′-Diaminobenzidine (DAB, Sigma-Aldrich, St. Louis, MO, USA) for 4 min at room temperature for 30 minUTES. The sections were counterstained with hematoxylin. All slides were randomly scored in ten microscopic fields at 400× magnification. Score 0 indicates that there are no immunopositive cells; score 1 indicates that there are between 1 and 25% immunopositive cells; score 2 indicates that there are between 26 and 50% immunopositive cells; score 3 indicates that there are between 51 and 75% immunopositive cells; and score 4 indicates that there are more than 75% immunopositive cells.

### 2.10. Measurement of Endothelial Function (Responses to Acetylcholine on Contraction of Norepinephrine (NE))

Ring segment rat aortas were cut (3 mm long) and hung vertically in an organ bath (organ bath system-820, MO, USA) containing 10 mL Krebs solution (composition in mM: NaCl 118; KCl 4.7; KH_2_PO_4_ 1.2; MgSO_4_ 1.18; NaHCO_3_ 25; D-Glucose 5.5, and CaCl_2_) at 37 °C and oxygenated with 95% O_2_ and 5% CO_2_. The ring of the aorta was equilibrated at a baseline of 1 g for 60 min and then exposed to Potassium Chloride 60 mM twice. Afterward, the ring of the aorta was exposed to Norepinephrine (1 µM), and, after a steady contraction, acetylcholine (1 µM) was added to assess endothelial cell function.

### 2.11. Histopathological Evaluation

Rat aortic tissue was fixed in 10% formalin buffer solution, dehydrated in ethanol, and embedded in paraffin. Furthermore, it was sliced at 4 µm and stained with Hematoxylin Eosin. Then, tissue was evaluated under a microscope to assess the aortic lumen diameter.

### 2.12. Statistical Analysis

The results are expressed as mean and standard deviation. The difference between groups was compared using a one-way analysis of variance (ANOVA), followed by Tukey’s post hoc test. The values are considered significant if *p* < 0.05.

## 3. Results

### 3.1. Dynamic Light Scattering Used to Characterize Fucoidan Nanoparticles

**Figure 1:**
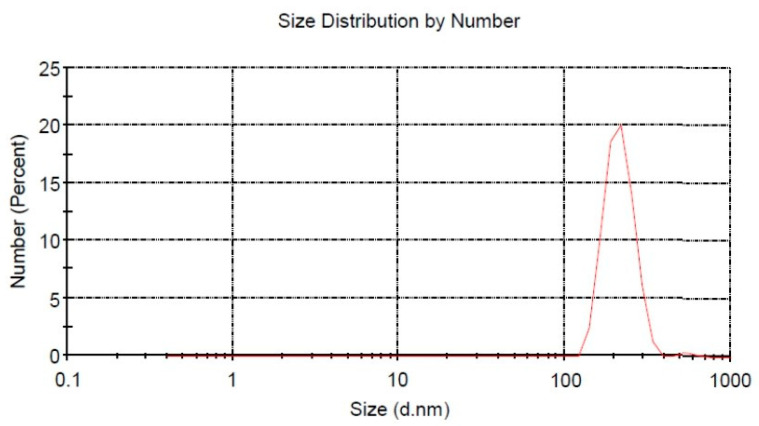
Fucoidan nanoparticle size distribution.

The particle size distribution of the Fucoidan nanoparticles was 267.2 ± 42.8 nm, which was analyzed by DLS, as shown in Figure 1.

### 3.2. Effect of Fucoidan Nanoparticles on ROS in Aortic Endothelial Cells of Diabetic Rats

**Figure 2:**
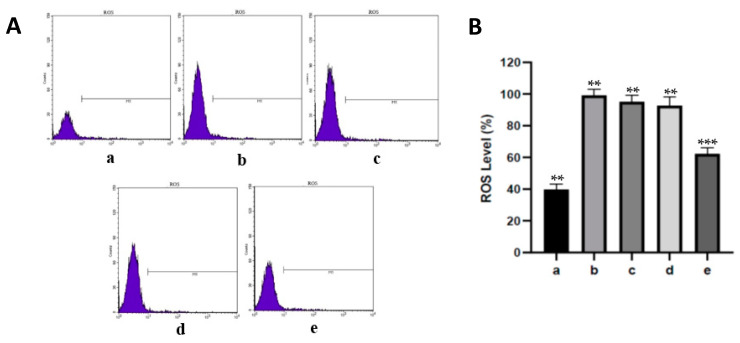
(**A**). Flow cytometry assay using DCFH-DA dye on fucoidan nanoparticles to assess effect on preventing oxidative stress. (**B**). The level of ROS in aortic endothelial cells was analyzed by flow cytometry. Control rats a; diabetic rats b; fucoidan nanoparticle rats, at doses of 75 c, 150 d, and 300 mg/kg BW e. **, *** The columns with different letters show significance between groups (*p* < 0.05).

The levels of ROS can be utilized as a marker for the presence of oxidative stress in cell damage. ROS levels of aortic endothelial cells from various treatments can be seen in Figure 2. ROS levels in diabetic rats increased with a significant difference when compared with the control rats at *p* < 0.05. However, dose-dependent administration of fucoidan nanoparticles decreased the level of ROS in the aortic endothelial cells of diabetic rats. Only the administration of fucoidan nanoparticles at a dose of 300 mg/kg BW significantly decreased the levels of ROS in the aortic endothelial cells of diabetic rats.

### 3.3. Fucoidan Nanoparticles’ Effects on the Levels of SOD and GPx in Diabetic Rats’ Aortas

Table 1 shows the SOD and GPx levels in aorta tissue from the various treatments. SOD and GPx are endogenous antioxidant enzymes that scavenge free radicals resulting in the inhibition of oxidative stress. In rats injected with streptozotocin, levels of SOD and GPx in the aortic tissue were decreased, which was significantly different when compared with the control rats, *p* < 0.05. On the other hand, the administration of fucoidan nanoparticles, depending on the dose, increased SOD and GPx levels in the aortic tissue of diabetic rats. Administration of fucoidan nanoparticles, only at a dose of 300 mg/kg BW, was significantly able to increase levels of SOD and GPx in the aortic tissue of diabetic rats, *p* < 0.05.

### 3.4. Effect of Fucoidan Nanoparticles on the Expression of Nrf2 in Diabetic Rats’ Aortas

**Figure 3:**
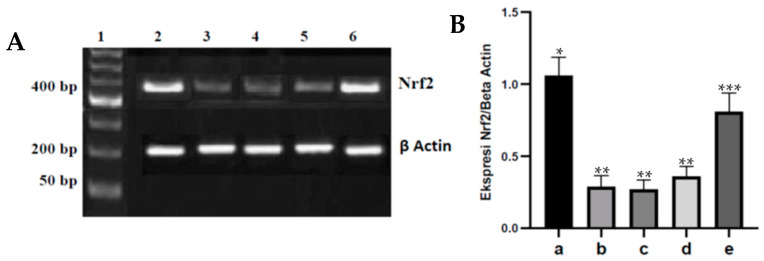
(**A**). Fucoidan nanoparticles’ effect on Nrf2 expression in aortic tissue of various treatments. (**A**). Nrf2 expression in aortic tissue: marker (1); control rats (2); diabetic rats (3); fucoidan nanoparticles at doses of 75 (4), 150 (5), and 300 mg/kg BW (6). (**B**). Fucoidan nanoparticles’ effect on Nrf2/β actin in aortic tissue: control rats a; diabetic rats b, Fucoidan nanoparticles at doses of 75 c, 150 d, and 300 mg/kg BW e. *, **, *** Columns with different letters statistically differ at *p* < 0.05.

The regulation of cellular antioxidants by Nrf2 plays a role in the defense against oxidative stress. The Nrf2 expression in aortic tissue from the various treatments is shown in Figure 3. Diabetes can significantly decrease Nrf2 expression in aortic tissue when compared with the control rats, *p* < 0.05. Meanwhile, dose-dependent administration of fucoidan nanoparticles increased Nrf2 expression in the aortic tissue of diabetic rats, and only a dose of 300 mg/kg BW significantly increased Nrf2 expression when compared to diabetic rats, *p* < 0.05.

### 3.5. Fucoidan Nanoparticles’ Effect on NO Levels in Endothelial Cells of Diabetic Rats’ Aortas

NO compounds can cause vasodilation and act as a regulator of vascular smooth muscle tone through the activation of soluble guanylate cyclase. The results of NO levels in aortic tissue from the various treatments are presented in Table 2. The diabetic rat group showed significantly decreased NO levels in the endothelial cells of the aorta when compared with the control rats, *p* < 0.05. However, depending on the dose, the administration of fucoidan nanoparticles increased the NO levels in the endothelial cell of the diabetic rats’ aortas, and only at a dose of 300 mg/kg BW. This shows how fucoidan nanoparticles are significantly able to increase NO levels in the endothelial cells of diabetic rats’ aortas, *p* < 0.05.

### 3.6. Effect of Fucoidan Nanoparticles on the Expression of cGMP in Diabetic Rats’ Aortas

**Figure 4:**
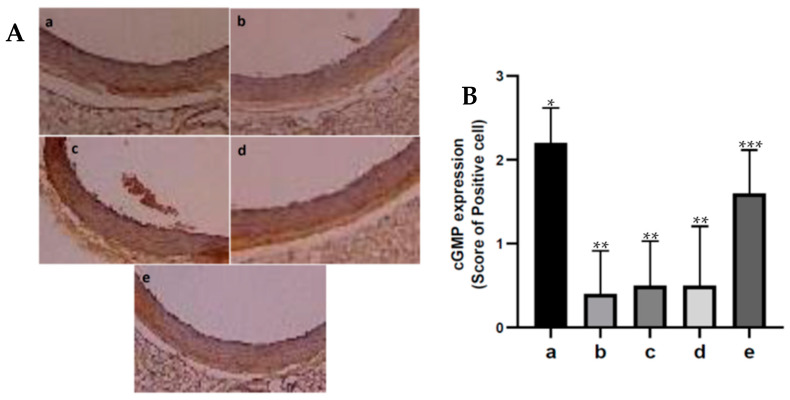
(**A**). Immunohistochemistry of the expression of cGMP in aortic tissue of diabetic rats: control rats a; diabetic rats b; fucoidan nanoparticles at doses of 75 c, 150 d, and 300 mg/kg BW e. (400×). (**B**). The number of cGMP-expressing cells that are immunoreactive. *, **, *** The columns with different letters show significance between groups (*p* < 0.05).

Cyclic GMP in vascular smooth muscle can cause vasodilation. The results of the immunohistochemistry testing of cGMP expression in aortic tissue from the various treatments are shown in Figure 4. In the diabetic rat group, cGMP production in aortic tissue decreased, which was significantly different when compared with the control rat group, *p* < 0.05. Meanwhile, diabetic rats administered with fucoidan nanoparticles increased cGMP production in the aortic tissue. Only a dose of 300 mg/kg BW significantly increased cGMP production when compared with diabetic rats, *p* < 0.05.

### 3.7. Fucoidan Nanoparticles’ Effect on Response Relaxation of Ach in Diabetic Rats’ Aortas

**Figure 5:**
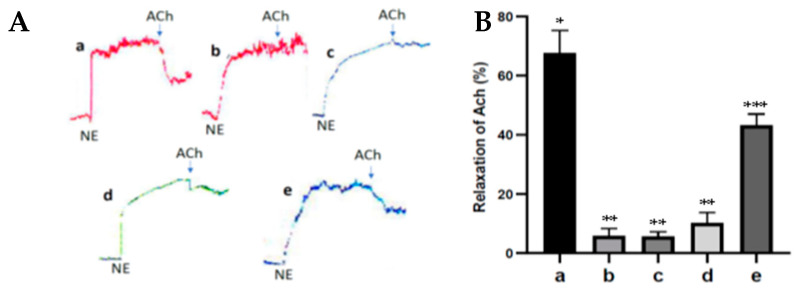
(**A**). The response of 1 µM Ach relaxation to 1 µM NE contractions from various treatments in rats’ aortas: control rats a; diabetic rats b; fucoidan nanoparticles at doses of 75 c, 150 d, and 300 mg/kg BW e. (**B**). The relaxation of Ach to NE contractions expressed in %. *, **, *** The columns with different letters show significance between groups (*p* < 0.05).

The acetylcholine smooth muscle relaxation response (vasodilation) can be used as a marker for endothelial cell damage. Figure 5 shows the response of Ach relaxation to NE contractions from the various treatments in the rats’ aortas. In the diabetic rat group, the inhibition of the relaxation response of 1 µM Ach to 1 µM NE contractions on the aorta within endothelial cells was significantly different, and the relaxation response was lower when compared to the control rats, *p* < 0.05. On the other hand, administration of fucoidan nanoparticles significantly increased the relaxation response of 1 µM Ach to 1 µM NE contractions on the aorta within endothelial cells of diabetic rats, *p* < 0.05. These results indicate that the administration of fucoidan nanoparticles can prevent aortic endothelial cell dysfunction in diabetic rats.

### 3.8. Fucoidan Nanoparticles’ Effect on Diameter of Aortic Lumens of Diabetic Rats

**Figure 6:**
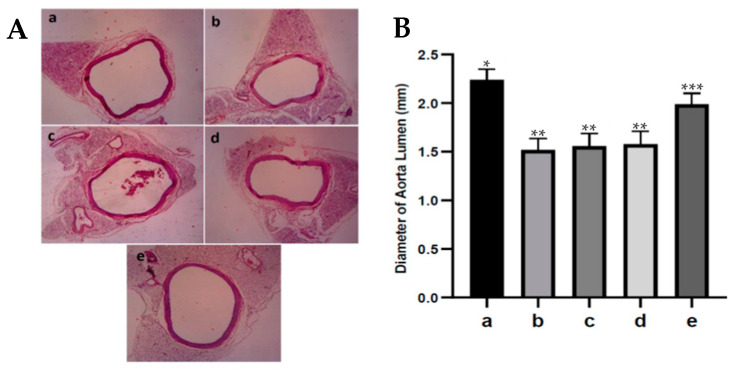
(**A**). The diameter of the aortic lumen from various treatments: control rats a; diabetic rats b; fucoidan nanoparticles at doses of 75 c, 150 d, and 300 mg/kg BW e. (400×). (**B**). The diameter of aortic lumens expressed in mm. *, **, *** The columns with different letters show significance between groups (*p* < 0.05).

An increase or decrease in the diameter of the aortic lumen indicates vasodilatation or vasoconstriction in the aortic vessels. The diameter of the aortic lumen from the various treatments can be seen in Figure 6. Narrowing of the aortic lumen (vasoconstriction) occurred in the group of diabetic rats, which was significantly different and had a lower diameter than the control rats, *p* < 0.05. In diabetic rats, administering fucoidan nanoparticles increased the diameter of diabetic rats’ aortic lumens, and only at a dose of 300 mg/kg BW. The fucoidan nanoparticles significantly increased the diameter of the diabetic rats’ aortic lumens, *p* < 0.05. These results show that the administration of fucoidan nanoparticles can prevent the narrowing of the aortic lumen (vasoconstriction) in diabetic rats.

## 4. Discussion

The progress of nanotechnology is quite rapid and the effectiveness of nanomaterials is extraordinary, so much so that nanotechnology is a new solution for the treatment of diabetes complications. Fucoidan nanoparticles are made into nano-size by the ball milling method, a technique that is widely used recently [26,27,28]. Size characterization of fucoidan nanoparticles was carried out using DLS, which showed a size distribution of 201.8 ± 14.6 nm. Fucoidan nanoparticles are considered an alternative solution to increase solubility, absorption, and bioavailability, as well as to increase their protective effect on complications of diabetes mellitus, especially in the dysfunction and damage of aortic endothelial cells. Oxidative stress has a major role in causing diabetes complications. Oxidative stress can increase the expression of inflammatory cytokines, which are both a consequence and a trigger for diabetes complications. Oxidative stress can occur due to the excessive formation of ROS and lower endogenous antioxidant defense systems [2,6,15].

In the diabetic rat model, STZ increases ROS, and induces oxidative stress, which plays an important role in endothelial cell dysfunction. Therefore, it has been necessary to investigate several parameters of oxidative stress and endothelial cell dysfunction, such as MDA, SOD, GPx, Nrf2, NO, cGMP, and the relaxation response of Ach in diabetic rat tissue [9,20,21]. STZ inhibits the secretion of insulin in the Langerhans islet beta cell, which leads to hyperglycemia complications such as endothelial cell dysfunction. The purpose of this research was to prove the antioxidant activity of fucoidan nanoparticles is able to protect against aortic endothelial cell dysfunctions in diabetic rats.

An increase in the amount of ROS in the body can cause oxidative stress and lipid peroxidation, namely the reaction of ROS with polyunsaturated fatty acids (PUFA) in cell membranes, which will produce MDA (Malondialdehyde) products. MDA can be used as a marker for the presence of free radicals, which can cause cell or tissue damage. The higher the level of MDA in the tissue, the higher the degree of tissue damage [2,6,7]. Our results showed that oral administration of fucoidan nanoparticles inhibited the increase in ROS levels in diabetic rat aortic tissue, which was significantly different when compared to the streptozotocin group at *p* < 0.05. Antioxidant compounds from fucoidan are able to scavenge and neutralize free radicals, thereby preventing an increase in MDA levels and the occurrence of oxidative stress. The antioxidant termination reaction of fucoidan can occur by capturing hydroxyl radicals (*OH) in the peroxidation reactions of lipids, proteins, or other molecules in the cell membranes so that the increase in MDA levels can be inhibited and cell damage can be avoided [24,25]. Fucoidan can also inactivate protein kinase C (PKC), causing the expression of various genes such as TGF-β, PAI-1, NF-κB, and NAD(P)H oxidase to decrease so that the production of ROS and MDA also decreases [29,30]

SOD and GPx are antioxidants that play an important role in tackling free radicals, namely superoxide anions and hydroxyl groups. SOD is present in almost all cells and is the first line of defense against ROS. SOD converts superoxide ions (O_2_^−^) to Hydrogen Peroxide (H_2_O_2_). Because Hydrogen Peroxide still reacts with other ROS, it needs to be degraded by other antioxidants, namely GPx. GPx detoxifies H2O2 by converting it into water and oxygen molecules. SOD and GPx work synergistically to support each other’s defenses against ROS. In addition, SOD and GPx inhibit oxidative stress, which is one of the diabetes biomarkers, the levels of which have been extensively examined in various diabetes complication studies [31,32]. In the results of this study, administering fucoidan nanoparticles inhibited the reduction in SOD and GPx levels in the aortic tissue of diabetic rats. Fucoidan is a polysaccharide substance that can inhibit the oxidation of important molecules such as proteins, lipids, and DNA caused by free radicals; they do so by donating electrons, so that they can inhibit the formation of free radicals and prevent oxidative stress, which in turn can inhibit vascular aortic damage, especially in endothelial cell dysfunction in diabetic complications [22,33,34]. The same results have been proven by Uslu et al. (2018), who reported that the administration of the antioxidant Cinnamomum cassia extract can inhibit the decrease in SOD and GPx levels of aortic tissue in rats injected intraperitoneally with streptozotocin. Wardani et al. (2022) also reported that the administration of fucoidan nanoparticles displayed antioxidant activity and inhibited the decrease in SOD and GPx levels in the kidney tissue of diabetic rats.

Cellular antioxidant regulation by Nrf2 plays a role in defense against oxidative stress. Nrf2 activity and expression decrease in diabetes caused by oxidative stress, which can weaken the induction of genes encoding antioxidants, resulting in decreased production of endogenous antioxidants such as SOD, GPx, and catalase [12,35]. Several researchers reported that streptozotocin can cause oxidative stress that occurs due to an imbalance between increased ROS production and decreased antioxidant capacity. This increase in ROS will cause cysteine damage in Keap1, disrupt the Keap1-Cul3 ubiquitination mechanism and mediate Nrf2 degradation by the 26S proteasome so that Nrf2 has a short half-life, which can result in a decreased expression of Nrf2 [13,36]. In rats injected with streptozotocin, administration of fucoidan nanoparticles orally inhibited the decrease in Nrf2 expression in the aortic tissue of diabetic rats. This is because fucoidan has a strong antioxidant effect and is a polysaccharide that has an OH group that can react with hydroxyl free radicals (∗OH), resulting in the inhibition of oxidative stress that can lead to an increase in Nrf2 expression. In addition to that, the strong antioxidant effect of fucoidan is able to reduce ROS so that cysteine damage does not occur in Keap1, which further inhibits the Keap1-Cul3 ubiquitination mechanism and Nrf2 degradation and can cause an increase in Nrf2 expression. The same results were reported that administration of the antioxidant fucoidan could inhibit oxidative stress and inhibit the decrease in Nrf2 expression in liver tissue induced by acetaminophen [14]. Administering the antioxidant chitosan nanoparticles orally to diabetic rats can also inhibit oxidative stress and inhibit the decrease in Nrf2 expression in heart tissue [15].

Nitric oxide (NO) is an endothelium-derived relaxing factor (EDRF) that under physiological conditions is released from endothelial cells in response to mechanical stimulation, acetylcholine, and increased intracellular calcium. NO compounds can cause vasodilation and act as a regulator of vascular smooth muscle tone through the activation of soluble guanylate cyclase [21,37]. The results of this study indicated that the administration of fucoidan nanoparticles orally increased eNOS expression and NO levels of aortic tissue in diabetic rats. It has been reported that antioxidants can be used to inhibit vascular endothelial cell dysfunction in diabetes by inhibiting ROS production so as to inhibit lipid peroxidation and prevent oxidation of cell membrane lipids, which can reduce MDA levels and increase SOD and GPx expression by inhibiting the reduction of Nrf2 expression, further inhibiting endothelial cell damage. This causes eNOS to remain active in producing NO, which causes vasodilation of the blood vessels [4,5]. Fucoidan has an activity to scavenge excessive superoxide anion free radicals, so that oxidation of the important cofactor tetrahydrobiopterin (BH4), which is required for the formation of eNOS, does not occur and can further increase NO production. Yeoh et al. (2021) reported the same results, which proved that giving the antioxidant propolis from Stingless Bees (Heterotrigona Itama) could increase eNOS expression and NO levels in aortic endothelial cells of diabetic rats.

Endothelial cells produce NO which will diffuse into the blood vessels’ smooth muscle cells and activate the soluble guanylate cyclase (sGC) enzyme, which can lead to increased formation of cyclic GMP from its GTP precursor. Cyclic GMP stimulates the relaxation of the smooth muscles of blood vessels so that vasodilation will occur [38]. Our results of this research show that oral administration of fucoidan nanoparticles increased cGMP expression in aortic tissue in diabetic rats. Administering exogenous antioxidants can increase NO formation and cGMP production so that aortic vasodilation can occur [20,21]. The results of this study indicate that the antioxidant activity of fucoidan nanoparticles with a scavenger can inhibit the reaction of superoxide ions with NO so that peroxynitrite which is toxic to endothelial cells is not formed, therefore, fucoidan can increase the formation of NO and cGMP. Yeohi et al. (2019) proved that administration of the antioxidant from Stingless Bees could increase endothelium-dependent relaxation of aortic smooth muscle by increasing the formation of NO and cGMP.

Endothelial cells actively regulate the relaxation and contraction of vascular smooth muscle under physiological and pathological conditions. Ach can cause relaxation of the smooth muscle of the aortic blood vessels (vasodilation) depending on the presence of endothelial cells because the administration of Ach can activate eNOS resulting in an increase in NO formation in endothelial cells, which in turn can activate soluble guanylate cyclase and increase the formation of cGMP that can cause vasodilation. Ach can be used to prove the occurrence of endothelial cell dysfunction. In diabetes, there is an inhibition of the Ach relaxation response due to endothelial cell dysfunction [19,20]. The results of this study show that the antioxidant activity of fucoidan nanoparticles increased the ACh relaxation response to norepinephrine contractions in the aortic blood vessels of diabetic rats. This is because the fucoidan nanoparticles work by donating their electrons so they can inhibit the formation of free radicals, increase the expression of SOD and GPx, and inhibit oxidative stress, which contributes to inhibiting the aortic endothelial cell dysfunction of diabetic rats. Furthermore, it can increase the production of NO and cGMP, so can cause aortic vasodilation and can increase the diameter of the aortic lumen. The same results also occur in the administration of propolis antioxidant from Heterotrigona itama, which can increase the relaxation response of acetylcholine to contraction of phenylephrine in the aorta of diabetic rats and works by increasing the production of NO and cGMP [20].

An increase or decrease in the diameter of the aortic lumen indicates vasoconstriction or vasodilation of the aortic blood vessels, which can cause narrowing or widening of the diameter of the aortic lumen [17,38]. Administration of fucoidan nanoparticles increased the diameter (dilation) of the diabetic rats’ aortic lumen. It has been reported that fucoidan has a strong antioxidant effect that can inhibit the process of lipid peroxidation because it can capture ROS in diabetic rats, thereby preventing endothelial cell damage; in addition, the eNOS remains active in converting L-arginine to NO. This NO can activate soluble guanylate cyclase in producing cGMP and cause an increase in the diameter (dilation) of the aortic lumen in diabetic rats.

Antioxidants are one option to prevent endothelial cell dysfunction in diabetes complications associated with oxidative stress. A number of studies have focused on the use of antioxidants to prevent diabetes complications such as neuropathy, retinopathy, nephropathy, hepatopathy, atherosclerosis, and vascular endothelial cell dysfunction. However, whether antioxidants have preventive and/or therapeutic value remains to be conducted in clinical trials on diabetics.

## 5. Conclusions

The results of this study can be concluded that fucoidan nanoparticles are natural-product antioxidants that can be used to prevent damage to aortic endothelial cells in the complications of diabetes, whose mechanism of action is through decreased levels of ROS and MDA, as well as increased expression of Nrf2, SOD, and GPx levels. This results in increased NO production and cGMP, which can increase the lumen diameter (vasodilation) of the diabetic rat aorta. Henceforth, it is necessary to study the antioxidant activity associated with inflammation and apoptosis in preventing endothelial cell dysfunction in diabetes complications.

## Figures and Tables

**Table 1:** Fucoidan Nanoparticles Effect on SOD and GPx Levels in aortic tissue from various treatments.

Group	SOD Levels (U/mg Protein) X ± SD	GPx Levels (U/mg Protein) X ± SD
Control Rats	5.98 ^a^ ± 0.43	49.0 ^a^ ± 2.6
Diabetic Rats	2.76 ^b^ ± 0.29	29.0 ^b^ ± 2.4
Fucoidan nano 75 mg/kg bw	2.70 ^b^ ± 0.24	28.3 ^b^ ± 1.7
Fucoidan nano 150 mg/kg bw	2.90 ^b^ ± 0.25	30.8 ^b^ ± 1.8
Fucoidan nano 300 mg/kg bw	3.88 ^c^ ± 0.35	35.6 ^c^ ± 2.1

^a–c^ The different superscript in each column show significantly diference between the means (*p* < 0.05).

**Table 2:** Fucoidan nanoparticles effects on NO levels aortic tissue of various treatment.

Group	NO Level Aorta (nmol/mg) X ± SD
Control Rats	1.47 ^a^ ± 0.21
Diabetic Rats	0.41 ^b^ ± 0.37
Fucoidan Nano Rats 75 mg/kg bw	0.39 ^b^ ± 0.31
Fucoidan Nano Rats 150 mg/kg bw	0.42 ^b^ ± 0.26
Fucoidan Nano Rats 300 mg/kg bw	0.94 ^c^ ± 0.37

^a–c^ The different superscript in each column show significantly diference between the means (*p* < 0.05).

## Data Availability

Data are presented within the article.
